# Depression and Recovery of Daily Life Autonomy in Patients With Stroke Receiving Home Rehabilitative Treatment

**DOI:** 10.4021/jocmr2010.02.250w

**Published:** 2010-02-15

**Authors:** Maria Concetta Cataldo, Alfonso Accursio, Maria Luisa Calcara, Giuseppe Caputo, Francesca Dal Maschio, Pasquale D'Antoni, Giuseppe Lima, Silvia Pirrotta, Giovanna Rizzo, Antonino Russo, Vincenzo Salerno, Caterina Mammina

**Affiliations:** a Geriatric Assessment Unit of Integrated Home care Health District 10, ASP6, Palermo, Italy; b Department of Sciences for Health Promotion G. DAlessandro, University of Palermo, Italy

## Abstract

**Background:**

The object of this study was to evaluate the prevalence of post stroke depression and its possible role as a predictive negative factor in patients receiving home rehabilitation treatment.

**Methods:**

We analyzed 103 patients with stroke by correlating comorbidities, clinical and blood test parameters and characteristics of the lesion with depression class identified according to the Hamilton scale and the outcome of the rehabilitation program.

**Results:**

A significant association between hypertension and post-stroke severe depression emerged in the female patients.

**Conclusions:**

Since the literature offers conflicting data, our results may contribute a stimulus for further studies.

**Keywords:**

Home care; Rehabilitation; Stroke; Depression; Hypertension

## Introduction

Stroke is the first cause of disability in Western countries and depression is the most frequent psychiatric disorder in patients with stroke. According to a recent systematic review of the literature post-stroke depression may affect up to 33% of these patients, with very wide fluctuations depending on whether the studies were based on hospital cases or population cases. Many studies show that between 30 and 50% of patients develop post-stroke depression in the early stages following the acute event and this seems to increase risk of mortality in the short, medium and long term [1,2].

Since subjects who have suffered an acute cerebral vascular event are very often multi-problem patients, depressive attitudes are often underestimated. In these cases, diagnosis of depression may be very challenging because patients complaints frequently refer to non specific somatic symptoms which are common in many chronic diseases affecting the elderly [3]. Indeed, according to recent reports, it is highly probable that unexplained somatic symptoms at a first clinical assessment, are actually parts of a depressive framework [4,5]. Even though the impact of post-stroke depression on the functional outcome of these patients has generated some interest, literature on its influence on the attentive functions and, consequently, on post-rehabilitation recovery, is limited. It is known that the natural history of stroke is positively influenced by rehabilitation which should be started as soon as possible. The quality of recovery is also determined by the type of cognitive processes that are being activated. Rehabilitation techniques are based on the assumption that the subjects may recover their lost or impaired ability or learn to reorganize their motor behaviour in relation to the residual functions surviving to the injury. Everything is then influenced by the ability or willingness to learn and save what is being learned [6,7]. Therefore, it is obvious that the identifying factors affecting the patients recovery may facilitate the prognosis quod valetudinem and the achievement of rehabilitation target.

The aim of our study was to measure whether and in what measure depression negatively affected the process of recovery of patients with stroke receiving home care. Moreover, given the frequent presence of post-stroke depression and its negative influence on the outcome of the rehabilitative treatment, we investigated possible associations between risk factors present in the acute phase of the illness and the onset of depression after the stroke.

## Patients and Methods

One hundred and three patients were recruited in the study at the Geriatric Assessment and Integrated Home Care (ADI) Unit of District 10, ASP 6, Palermo, Italy. The ADI Unit includes an internal medicine specialist, a physiatrist, a social worker, a registered nurse, a physiotherapist and a visiting healthcare nurse and it collaborates with a geriatrician and a psychiatrist of the District Health Clinic. Data was collected regarding socio-demographic characteristics and the presence of vascular risk factors and comorbilities, such as hypertension, diabetes mellitus, dyslipidemia, coronary disease or previous stroke. Etiology and location of the cerebral lesions were also registered.

All patients included in the study were assessed through a multidimensional clinical approach both at admission and at the time of discharge. At first, the social worker through an interview with family members evaluated the home care organization of the patient. Then the multidisciplinary assessment was carried out by collecting anamnestic data and the clinical examination.

The following scales were used: the Barthel index for activities of daily living, the Barthel mobility index for the functional evaluation and the Short Portable Mental Status Questionnaire (SPQM) for the cognitive evaluation [8,9]. Depression was diagnosed according the DSM IV diagnostic criteria [10,11] using, among the several scales developed and validated over the years to identify subjects likely to have depressive symptoms, the Hamilton Rating Scale for Depression (HDRS) [12,13]. This scale consists of 21 items that focus on the typical symptoms of depression and are able to identify its degree of severity. The assessment of depression in the HDRS scale classifies subjects with stroke in non-depressed (0 - 7), mildly depressed (8 - 17), moderatly depressed (18 - 24) and severly depressed (over 25).

The home rehabilitation program provided three one hour sessions a week (extensive rehabilitation) with therapeutic exercises designed to obtain the recovery of functional movement. To evaluate the outcome of rehabilitation treatment, the Barthel scales of daily living activities and mobility were used.

In contrast to most studies that have been conducted until now and in which a reliable assessment of depressive symptoms with the actual post-stroke assessment scales had been considered impossible or unreliable with aphasic patients, in this study were included aphasic patients if they were able to answer to the Hamilton Rating Scale for Depression (HDRS) with simple yes or no answers or with gestures.

The statistical analysis was performed with the software EpiInfoTM 3.5 (CDC, Atlanta, GA, USA). Due to the relatively small number of cases, for the statistical analysis the HDRS classes were collapsed into two, mild to moderate (≤ 17) and severe (≥ 18). The descriptive analysis was performed by calculating the mean (standard deviation) and frequencies and the significance of differences was assessed by one-way ANOVA test or Kruskall-Wallis, when appropriate, or with the chi-square test or the exact test of Fisher, respectively. The associations between the variables under examination were evaluated using contingency tables. The results of the statistical tests were considered significant for P values ≤ 0.05.

## Results

Sixty-two out of the 103 subjects under study were of female gender. The mean age of these patients was 76.6 ± 9.1 years with a significant difference by gender (F, 78.8 ± 8.5 vs. M, 73.1 ± 8.8, P = 0.001). The anamnestic data related to comorbilities, the clinical chemical parameters and the characteristics of the lesions are summarized in Table 1. Among the variables under analysis, only hypertension appeared significantly related to female gender (F, 90.3% vs. M, 68.3%, P = 0.003), whereas a positive anamnesis for a previous stroke was significantly more frequent in male gender (F, 24.2 vs. M, 46.3, P = 0.01). Twenty-nine subjects were aphasic. Aphasia appeared significantly associated with female gender (F, 35.5% vs. M, 17.1, P = 0,02).

**Table 1:** Socio-Demographic, Anamnestic Characteristics and Etiological Subtype/Location of the Lesion in the 103 Patients Under Study

Characteristic	N (%)
Female gender	62 (60.2)
Education (years)	
< 8	91 (88.3)
≥ 8	12 (11.7)
Caregiver	
Household member	77 (74.8)
other	26 (25.2)
Hyperthension	84 (81.6)
Coronaropathy	15 (14.7)
Diabetes	38 (36.9)
Dyslipidemy	23 (22.5)
Previous stroke	34 (33.0)
Location of the lesion	
Right emisphere	33 (32.0)
Left emisphere	56 (54.4)
other	14 (13.6)
Etiological subtype*	
aterotrombotic	55 (53.4)
cardioembolic	51 (49.5)
Small vessels disease	32 (31.1)

*The total is higher than 103, because some patients had a mixed subtype.

No patients were classified as non-depressed. The distribution included 20 patients (19.4%) in the major depression class and 83 (80.6%) in the mild to moderate class. The HDRS class did not appear significantly associated with age or gender or any socio-demographic variable, including age, sex and parental status, if any, of the caregiver.

**Figure 1:**
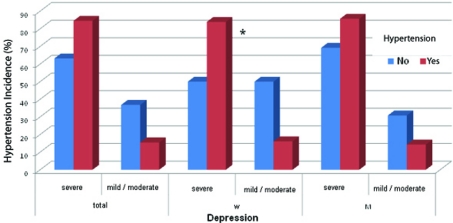
Incidence of hypertension according to gender and severity of depression. W: women; M: men. P = 0.02.

With regard to the anamnestic characteristics, location and type of the lesion, the only variable showing a statistically significant association with the attribution of the patient to the HDRS class was hypertension. Indeed, patients with hypertension were more frequently included in the HDRS severe class than the non-hypertensive ones (Fig. 1). The subsequent stratification by gender showed that the association was statistically significant in the female gender but not in the male gender, although within the male patients emerged also an asymmetrical distribution with a higher frequency of hypertension among subjects included in the major depression class.

Finally, among our patients, attribution to the mild/moderate or, alternatively, severe HDRS class did not seem to affect the outcome of treatment, since there were no statistically significant differences between the Barthel index means after stratifying by class the results of the patients assessed at the beginning and at the end of the period of treatment.

## Discussion

The onset of depressive symptoms is a frequent complication of stroke, associated with a worse functional prognosis and limited success of rehabilitation treatment. Given the high prevalence of this disorder and its consequences on the outcome, it is important to study the predictive factors and the variables that may influence its development and implications. The Hamilton scale used in the study for the assessment of depression proved to be extremely sensitive in highlighting even small deflections of mood so that virtually all patients in the post-stroke period were somewhat affected by depression. This finding led us to compare patients with mild depression vs. patients with severe depression and this may have weakened some of the associations under study.

It is well known that the participation of the family plays a key role in the overall management of the home care patient with sequelae of stroke. In our study however the analysis of age, sex and parental status of the caregiver showed no association with the severity of depression after stroke. Similarly, no statistically significant association emerged from the analysis of the socio-economic condition of the patients (age, marital status, instruction level, former occupation). Likewise, most of the comorbilities, the patient's cognitive status and the aetiology of stroke showed no association with depression.

In previous studies, a controversial issue has been the possible influence of the site of brain damage on the later development of depression. Systematic reviews did not support this hypothesis and in our study we also observed no differences in the frequency of mild/moderate or severe depression in relation to the location of brain damage [14,15].

Our study has shown a highly significant association between hypertension and severe depression. Several studies [16-20] have shown a higher prevalence of clinical depression and severe depressive symptoms in patients with chronic diseases including hypertension. Moreover, a recent meta-analysis that analyzed the association between chronic illnesses and depression evidenced an association between hypertension and depression although the correlation was not highly significant [21]. It seems appropriate, here, to briefly recall the biochemical mechanisms responsible for depression and their link with hypertension. Particularly interesting is the activation of the sympathetic nervous system and the hypothalamic-pituitary-adrenal axis, which determines an increase in heart rate, peripheric vascular resistance, secretion of cortisol, catecholamines, glucose and insulin. All these mechanisms may contribute to the onset or the progression of hypertension [22]. The activation of the hypothalamic-pituitary-adrenal axis plays also a central role in stimulating endothelial growth factors and, consequently, on atherogenesis inflammation-mediated. Some studies have documented in patients with severe depression an elevated activity of the sympathetic nervous system with increased plasma levels of cortisol [3]. In addition to the direct effects on the vascular system, the activation of these biochemical pathways may be associated with an increased release of inflammatory cytokines, a hypothesis supported by the finding of elevated levels of C-reactive protein in these patients [23]. The relationship between hypertension and depression could evolve, therefore, through a series of biological reactions.

Integrating different professional skills to ensure the unity, fullness and coherence of the rehabilitative activities is undoubtedly a major challenge to warrant the quality of care delivered at home.

In our social setting, the motor-sensory rehabilitation of a patient who has suffered a cerebral ischemic event aims to prevent the immobilization syndrome, to facilitate the recovery of and/or to maintain correct posture and functional motions in order to achieve the highest possible degree of autonomy in activities of daily living with reintegration in social life and relations. It is always necessary to consider the patient in his mental and physical unity and complexity in order to implement an effective and efficient therapeutic program. It is always difficult for these patients to achieve the full recovery of previous functional abilities, so counselling is necessary in order to help both the patient and the family group to accept the new situation and to build a new balance. Depression should be considered as a part of the problems of post-stroke patients and clinical practice should pay more and more attention to it because it can be an important element in causing disability and a poorer quality of life. Indeed, depressed patients exhibit poor functional performances which are comparable to or worse than the performances of patients with chronic medical conditions. Therefore, early recognition of depressive symptoms in patients affected by stroke is generally considered to be a critical factor in order to promote a better quality of their life [24,25].

Currently, strategies for preventive therapy of depression are incompletely formulated. It would be more appropriate to focus on a better understanding of the neurobiology of depression and hypertension as a basis for developing a rational and effective therapy. Considerations deriving from our findings, though obtained from a limited number of patients, should represent a stimulus for more extensive investigations.

"It is not the flexing of the leg that we must seek, but the marching function and the march itself is only one element of normal human behaviour; in fact we must believe that whoever walks, eats and sleeps, he will also begin to smile... " Ueberschlag H.
